# *Pantoea stewartii* subsp. *stewartii* an Inter-Laboratory Comparative Study of Molecular Tests and Comparative Genome Analysis of Italian Strains

**DOI:** 10.3390/plants14101470

**Published:** 2025-05-14

**Authors:** Valeria Scala, Nicoletta Pucci, Riccardo Fiorani, Alessia L’Aurora, Alessandro Polito, Marco Di Marsico, Riccardo Aiese Cigliano, Eleonora Barra, Serena Ciarroni, Francesca De Amicis, Salvatore Fascella, Francesca Gaffuri, Andreas Gallmetzer, Francesca Giacobbi, Pasquale Domenico Grieco, Valeria Gualandri, Giovanna Mason, Daniela Pasqua di Bisceglie, Domenico Rizzo, Maria Rosaria Silletti, Simona Talevi, Marco Testa, Cosimo Tocci, Stefania Loreti

**Affiliations:** 1 Research Centre for Plant Protection and Certification (CREA-DC), Council for Agricultural Research and Economics, 00156 Rome, Italy; nicoletta.pucci@crea.gov.it (N.P.); riccardo.fiorani@crea.gov.it (R.F.); alessia.laurora@crea.gov.it (A.L.); alessandro.polito@crea.gov.it (A.P.); stefania.loreti@crea.gov.it (S.L.); 2 Sequentia Biotech, 08005 Barcelona, Spain; mdimarsico@sequentiabiotech.com (M.D.M.); raiesecigliano@sequentiabiotech.com (R.A.C.); 3 Phytopathological Laboratory, Campania Region, 80141 Napoli, Italy; eleonora.barra@unina.it; 4 Phy.Dia. srl Via San Camillo de Lellis, snc (DAFNE-UNITUS), 01100 Viterbo, Italy; info@phydia.eu; 5 ERSA Regional Agency for Agricultural Development—Plant Protection Service of Friuli Venezia Giulia Region—Phytopathology and Biotechnology Laboratory, 33050 Pozzuolo del Friuli, Italy; francesca.deamicis@ersa.fvg.it; 6 Regional Plant Protection Service, Liguria Region, 16129 Genova, Italy; salvatore.fascella@regione.liguria.it; 7 Laboratorio del Servizio Fitosanitario Regione Lombardia, V.le Raimondi 54, 22070 Vertemate con Minoprio, Italy; francesca_gaffuricnt@regione.lombardia.it; 8 Laimburg Research Centre, 39040 Auer, Italy; andreas.gallmetzer@laimburg.it; 9 Phytosanitary Laboratory—Plant Protection Service, Emilia-Romagna Region, via A. da Formigine n.3, 40128 Bologna, Italy; francesca.giacobbi@regione.emilia-romagna.it; 10 Research, Training and Advanced Service Area Plant Pathology Group Leader ALSIA—Metapontum Agrobios Research Center S.S. Jonica 106—Km 448,2, 75012 Metaponto, Italy; pasquale.grieco@alsia.it; 11 Fondazione Edmund Mach, Technology Transfer Centre, Unit of Laboratory of Phytopathological Diagnostics, 38098 San Michele all’Adige, Italy; valeria.gualandri@fmach.it; 12 Phytosanitary Laboratory of the Phytosanitary Sector and Technical-Scientific Services of the Piedmont Region, 10144 Torino, Italy; giovanna.mason@regione.piemonte.it; 13 Istituzione di Appartenenza: Veneto Region (IT), Plant Protection Service, 30123 Venezia, Italy; daniela.pasquadibisceglie@regione.veneto.it; 14 Laboratory of Phytopathological Diagnostics and Molecular Biology, Plant Protection Service of Tuscany, Via Ciliegiole 99, 51100 Pistoia, Italy; domenico.rizzo@regione.toscana.it; 15 Centro di Ricerca Sperimentazione e Formazione in Agricoltura (C.R.S.F.A.) “Basile Caramia”, 70010 Locorotondo, Italy; mariasilletti@crsfa.it; 16 Plant Protection Service Diagnostic Laboratory—AMAP Marche Region, 60027 Osimo Stazione, Italy; talevi.simona@assam.marche.it; 17 Agris Sardegna, Agricultural Research Agency of Sardinia, 09020 Ussana, Italy; mtesta@agrisricerca.it; 18 Laboratorio Fitopatologico Ufficiale ARSAC di San Marco Argentano (Official Phytosanitary Laboratory ARSAC of San Marco Argentano), 87018 San Marco Argentano, Italy; cosimo.tocci@arsac.calabria.it

**Keywords:** test performance study, *Pantoea stewartii* susb. *stewartii*, diagnosis, performance criteria, genome, sequencing

## Abstract

*Pantoea stewartii* subsp. *stewartii* (Pss) is a Gram-negative bacterium causing Stewart wilt, a severe disease in maize. Native to North America, it has spread globally through the maize seed trade. Resistant maize varieties and insecticides are crucial to mitigate the disease’s economic impact. Pss is a quarantine pest, requiring phytosanitary certification for the seed trade in European countries. Accurate diagnostic tests, including real-time PCR, are fundamental to detect Pss and distinguish it from other bacteria, like *Pantoea stewartii* subsp. *indologenes* (Psi), a non-quarantine bacteria associated with maize seeds. Population genetics is a valuable tool for studying adaptation, speciation, population structure, diversity, and evolution in plant bacterial pathogens. In this study, the key activities of interlaboratory comparisons are reported to assess diagnostic sensitivity (DSE), diagnostic specificity (DSP) and accuracy (ACC) for different real-time PCR able to detect Pss in seeds. The results of complete sequencing of Italian bacterial isolates are presented. This study enhances our understanding of molecular methods for diagnosing and identifying pathogens in maize seeds, improving knowledge of Pss genomes to prevent their spread and trace possible entry routes from endemic to non-endemic areas.

## 1. Introduction

*Pantoea stewartii* subsp. *stewartii* (Pss), a gram-negative bacterium, is the primary causal agent of Stewart wilt, a devastating disease affecting maize. Belonging to the *Erwiniaceae* family and *Pantoea* genus [1,2], it is listed as a quarantine pest in the part A of the EU Regulation 2072/2019 and in the A2 list by the European Plant Protection Organization (EPPO) [3]. Indigenous to North America, vectoring by *Chaetocnema pulicaria* Melsheimer and global spread have been facilitated through the trade of maize kernels [3]. Maize varieties resistant to Pss and the application of insecticides play a pivotal role in mitigating the economic impact of Stewart wilt disease in the United States [4]. Conversely, susceptible maize varieties face severe consequences, often succumbing to disease at the vulnerable seedling stage [4]. The symptoms of Stewart wilt disease are categorized into two main groups: wilt and leaf blight [5]. The wilt phase primarily affects young seedlings infected with Pss, leading to their eventual demise. Conversely, leaf blight symptoms tend to manifest in mature plants but typically do not result in plant death. According to the EPPO data base [6], Stewart’s wilt is present (few occurrences) in Russia, and is transient in Italy, Slovenia and Ukraine. The Italian Plant Protection Services carried out the surveillance of the Italian territory and notified interested parties of the presence of the causal agent of Stewart’s wilt disease of *Zea mays* in 2015, 2016, 2017, 2018, 2020, 2022, 2023 and 2024 (https://webgate.ec.europa.eu/europhyt, accessed on 28 March 2025) [7]. The *Pantoea* genus includes Pss, *Pantoea ananatis* (Pan), *Pantoea indologenes* (Psi), *Pantoea allii*, and *Pantoea agglomerans*; only Pss is a quarantine pest, and many countries impose phytosanitary restrictions requiring phytosanitary certification of seeds, “Pss-free” [1,8]. While numerous diagnostic tests exist for detecting Pss, only a few can accurately identify this quarantine bacterial pathogen without the risk of misidentification with Psi, the species taxonomically closest to it. Psi is not a regulated pest, and it occasionally appears in maize seeds of tropical or subtropical origin [9]. Among the available real-time PCR methods, only the test developed by Pal and colleagues [10] has demonstrated the capability to distinguish Pss from Psi [11]. Moreover, this method enables direct detection of Pss from maize seeds without the need for bacterial isolation, as highlighted by Dreo [11]. Considering the significance of Stewart’s wilt disease, efforts to validate and standardize diagnostic tests for Pss were undertaken as part of the EU project Valitest (grant agreement No. 773,139). Additionally, a previous Italian project Proteggo 1.4 and the European EURL-BAC project carried out activities aimed at deepening our understanding of this pathogen. Within the scope of these projects, CREA-DC yielded a novel diagnostic test Scala and colleagues [7] for detecting Pss in maize seeds with good parameters of sensitivity and specificity and sequenced several Italian strains to shed light on their possible origins. This concerted effort reflects the commitment to enhancing diagnostic capabilities and bolstering plant health safeguards against this destructive pathogen. In this context, our study focuses on two aspects to foster the understanding of Pss:

(i) Comparison of different molecular tests to detect Pss: real-time PCR Scala et al., (2023) [7], real-time PCR Tambong et al., (2008) [9] and real-time PCR Pal et al., (2019) [10] (hereafter named Scala, Tambong and Pal, respectively) through interlaboratory comparisons (ILC). ILC combines the results obtained from a Proficiency Test (PT) and a Test Performance Study (TPS) conducted on the same set of samples;
(ii) Complete sequencing of five bacterial isolates collected in Italy between 2015 and 2022, utilizing the Oxford Nanopore MinION technology alongside the Illumina methodology. The study concerned several key objectives: (a) sequencing and assembling Pss strains; (b) conducting genomic characterization studies to compare the isolates with existing data; and (c) performing a comparative analysis of the sequenced genomes.

This study lets us deepen our understanding of the molecular methods available for the diagnosis of the pathogen in maize seeds and improve the knowledge of the genomes of Pss for preventing the introduction and the spread of the pathogen and to trace possible entry routes of this pathogen from endemic areas to non-endemic areas.

## 2. Results

### 2.1. Proficiency Test (PT), Test Performance Study (TPS) and ILC (Interlaboratory Comparison)

#### 2.1.1. Sample Homogeneity and Stability Test

The results of homogeneity and stability (mid-term and long-term stability) tests for all samples are reported in Tables S1–S3. The results were in accordance with the phytosanitary status of the samples (healthy or spiked); the value of the maximum and minimum Cq values obtained for each sample, the mean Cq values, and the respective coefficient of variation (CV) values are reported. The results are in the acceptable range of variations for all the samples.

#### 2.1.2. Proficiency Test (PT)

All OLs presented datasets with concordant results for all control samples and were therefore deemed valid for data analysis (Table S4). The participant OLs used different DNA extraction protocols: 71%, the Qiagen DNeasy Plant Mini Kit; 12%, the NucleoSpin Microbial DNA Kit by Macherey-Nagel; 12%, the MaxWell16 by Promega; and 5%, the Plant DNA DS Mini Kit by OMEGA Biotek (Norcross, GA, USA). None of the laboratories gave inconclusive results. The method developed by Pal and colleagues [10] was the most widely used (94%), followed by Tambong and colleagues [9] (53%), the Loewe real-time PCR kit (Lucknow, India) (6%), the cPCR method with Ages primers as reported in PM 7/60(2) [3] (6%), the Loewe PCR kit (6%), and the cPCR method by Thapa and colleagues [12] (6%).

**Figure 1:**
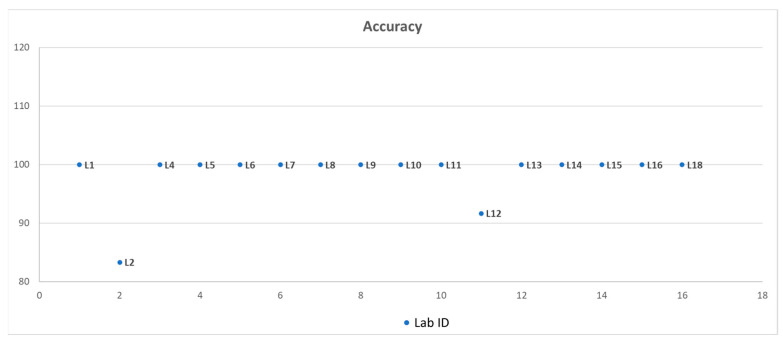
Percentage of accuracy achieved by each official laboratory (OL) participating in the PT activity. The *y*-axis shows the percentage of accuracy; the *x*-axis shows the number of OLs participating in the PT, indicated with an alphanumeric code (letter L and number).

The DSE was 100% for 16 OLs, whereas the L02 obtained 66.7% for the presence of two false-negative results with the samples S2 and S6. The DSP was 100% for 16 OLs, while the L12 obtained 83.3% for a false-positive result with sample S3. Fifteen laboratories achieved 100% ACC, while two laboratories had a lower level of ACC due to the presence of false negatives (Lab 02, 83.3%) and false positives (Lab 12, 91.7%) (Figure 1). All laboratories performed at least one of the methods listed in PM 7/60 (2) [3] and/or Pal and colleagues [10]. 

#### 2.1.3. Test Performance Study (TPS)

Among the OLs involved in the PT, some laboratories employed the same samples set to participate in the TPS and validate the Scala test [7] with two different Taq polymerases, grouped as Scala App and Scala Prom. The OLs gave concordant results for all control samples, and therefore the results were considered valid. None of the laboratories gave inconclusive results. The performance of each master mix used for Scala [7] is reported in Table 1. DSE, DSP and ACC were equal to 100% for both master mixes regardless of the DNA extraction method used. None of the OLs obtained false negative or false-positive results (non-concordant values) in detecting Pss with either of the master mixes used. In the absence of non-concordant data, the reproducibility value is 100% with both master mixes used.

#### 2.1.4. Interlaboratory Comparison (ICL)

To compare the most commonly used methods in PT, Pal [10] and Tambong [9], with Scala [7] used in TPS, the performance criteria of each test were analyzed (Table 1). DSE, DSP and ACC were all equal to 100% except for Tambong [9], where the DSE value was 83.3% due to the presence of false-positive results for sample S3, which consisted of a bacterial suspension of Psi.

### 2.2. De Novo Genome Assembly

For the strains CREA-DC 1990 (Pss_1990), CREA-DC 1870 (Pss_1870) and CREA-DC 1869 (Pss_1869), both long and short reads were produced. For CREA-DC 2061 (Pss_2061) and CREA-DC 2123 (Pss_2123), only short reads were available. Illumina reads passed through the trimming step, and results are reported in Table 2.

**Figure 2:**
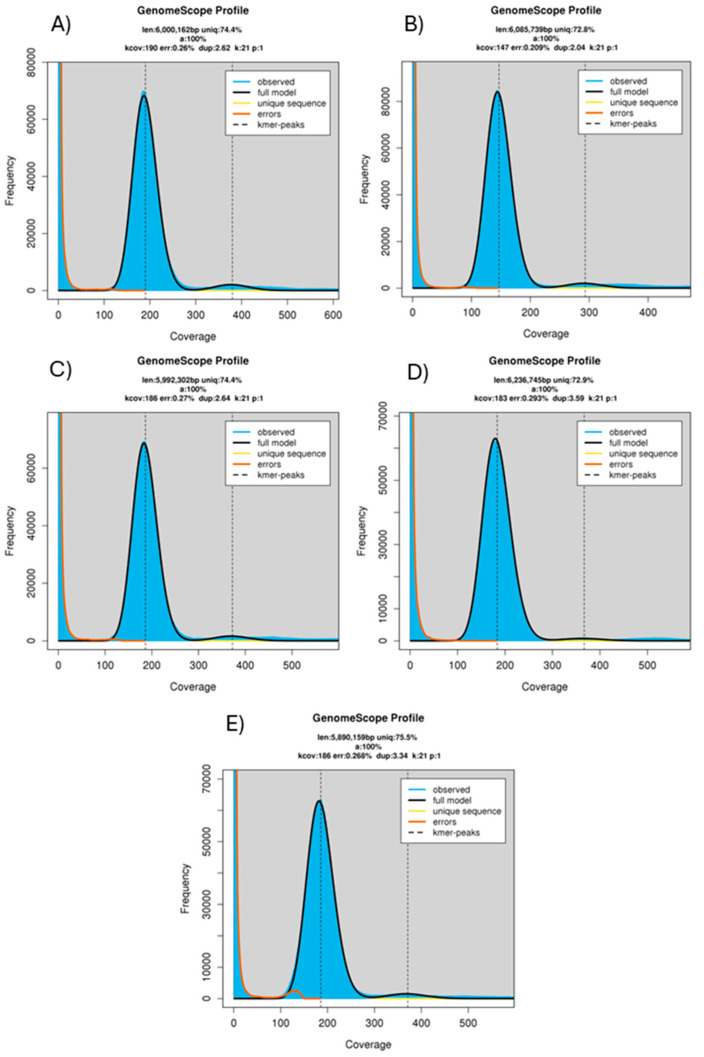
GenomeScope profiling for (**A**) Pss_1990, (**B**) Pss_1870, (**C**) Pss_1869, (**D**) Pss_2061, (**E**) Pss_2123. On the *x*-axis, coverage of k-mers is reported, while on the *y*-axis are reported the k-mer counts. On the top of the plot are reported: len (inferred total genome length), uniq (percent of the genome that is unique), het (overall rate of heterozygosity), kcov (mean k-mer coverage for heterozygous bases), err (error rate of the reads), dup (average rate of read duplications). Pss = *Pantoea stewartii* subsp. *stewartii*.

With the Illumina reads data, GenomeScope (http://genomescope.org/genomescope2.0/, 10 December 2024) was used to evaluate the genome size for the strains: 6,000,162 bp for Pss_1990 (Figure 2A), 6,085,739 for Pss_1870 (Figure 2B), 5,992,302 for Pss_1869 (Figure 2C), 6,236,745 for Pss_2061 (Figure 2D), 5,890,159 for Pss_2123 (Figure 2E). In each one of the plots in the figure, the following data are reported: inferred total genome length, percent of the genome that is unique, overall rate of heterozygosity, mean k-mer coverage for heterozygous bases, error rate of the reads, and average rate of read duplications.

The high-quality reads were used as inputs for the de novo assembly of each strain considering also the genome size estimation obtained in the previous step. The average size of the obtained assemblies was 5,310,337 bp, with a standard deviation of 115,894 bp. In Table 3, it is possible to check the general statistics for the produced assemblies.

The assembled genomes were finally annotated using the software Prokka. In Table 4, the number of genes for each strain is reported, with an average of 5573 loci and a standard deviation of 213.

**Figure 3:**
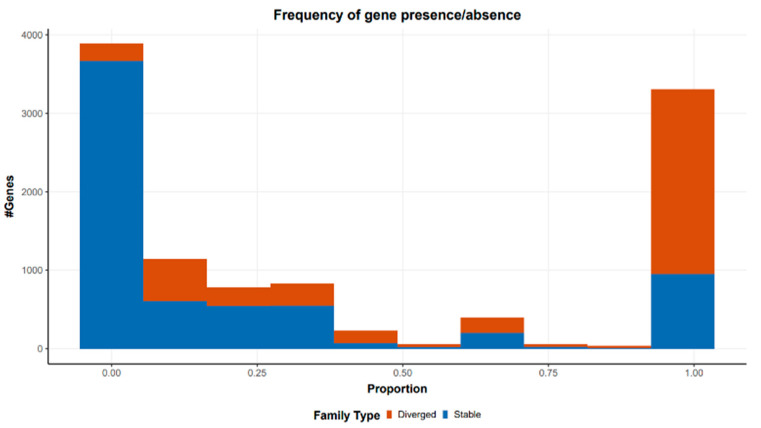
Distribution of gene presence/absence across the analyzed genomes. The *x*-axis shows the proportion of genomes in which each gene family is found, and the *y*-axis shows the number of gene families at each proportion. Gene families are considered stable (blue) when they have only a single allele at 98% amino acid identity and diverged (red) when they have >1 allele reflecting greater sequence variation. The presence/absence profile helps differentiate core genes, which tend to be both conserved and widely distributed, from accessory and variable genes, which often show reduced prevalence and increased sequence heterogeneity.

The MASH analysis allowed us to calculate the MASH distances between all the *Pantoea* strains (the freshly assembled and those downloaded from NCBI). Interestingly, all the new *Pantoea* strains are included in the same clade of other *Pantoea stewartii* subsp. *stewartii*, collected in Italy from the same Center of Research, while the other assemblies are included in the *Pantoea stewartii* subsp. *indologenes* clade. Finally, a pangenome analysis performed with the PIRATE toolbox allowed the identification of 10,705 gene families shared between the 53 analyzed genomes; 3021 loci identified as core loci (1%), while 254,435 were classified as non-core loci (99%) (Figure 3).

**Figure 4:**
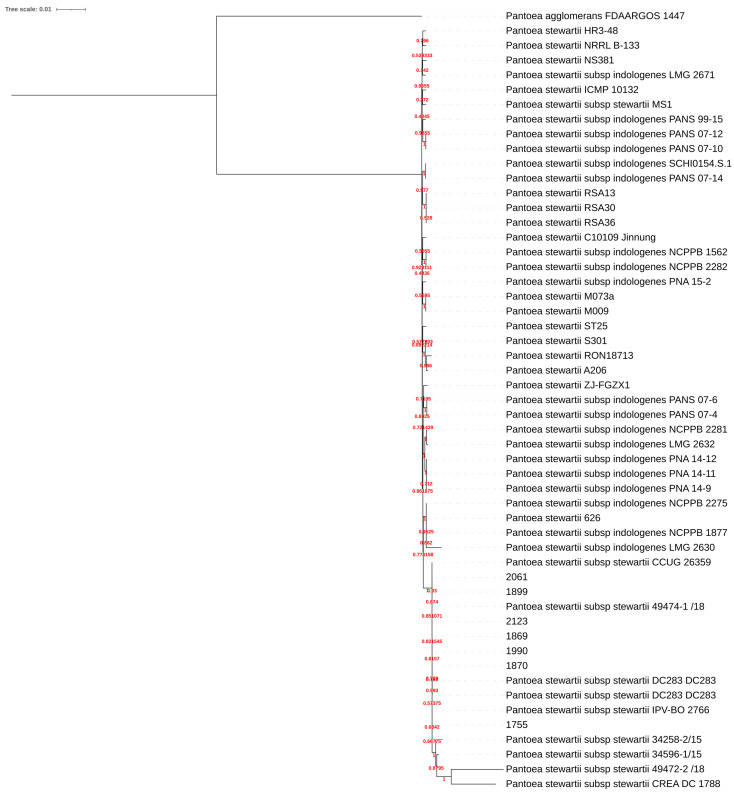
Species tree rooted on *Pantoea agglomerans*. Bootstrap values are reported in red.

Figure 4 reports the tree generated with Orthofinder and the raxml-ng algorithm. From the tree, it is possible to see that the newest assembled *Pantoea* proteomes are in the same clade of the Pss, while all the Psi cluster together in another clade.

## 3. Discussion

Climate change and globalization have contributed in recent decades to the emergence of epidemics caused by phytopathogenic bacteria affecting agricultural crops and have significantly impacted the distribution and diversity of plant pathogens worldwide [13,14]. International organizations (EFSA, EPPO, IPPC) and scientific research prioritize the development of prevention systems to avoid the introduction and spread of alien species. This involves enhancing the effectiveness of diagnostic tools and investigating the origins and pathways for the introduction of pests.

Following recent reports of Pss in Italy [7], an ILC was conducted among Italian OLs to assess their proficiency and compare various molecular diagnostic methods available for detecting this pathogen. The capability of OLs to detect the pathogen, coupled with a deeper understanding of the pathogen—which has not yet been established in Europe—is essential to prevent its introduction and spread.

Genome sequencing of strains isolated in Italy in recent years enhances our knowledge of the genomic diversity of Pss, providing valuable sequences to identify new targets and improve the analytical specificity of diagnostic tests.

Pss poses several challenges that significantly threaten maize crops. Originally from North America, the pathogen spreads worldwide through maize seeds. The impact of Stewart’s wilt is exacerbated in temperate climate conditions, such as those that have occurred in the EU in recent years due to the advance of climate change [15]. The insect vector, the main pathway of Pss dissemination in USA, is absent in EU. Pss can be also transmitted by infected seeds via commercial routes; seeds can be infected asymptomatically with a low bacterial load, making interception of the pathogen difficult. The current EPPO protocol [3] includes only some of the available molecular tests. In 2020, within the EU project Valitest (grant agreement N° 773,139), an interlaboratory study was organized to validate the tests described in the EPPO protocol [3] with newly available tests from the literature [11]. This activity highlights the performance of the assessed tests. In particular, Tambong [9] was found to be nonspecific, unable to distinguish Pss from Psi. Pal [10] and the conventional PCR by Gehring and colleagues [16] are specific for Pss. However, this protocol [16] has low analytical sensitivity and is not reliable for detecting Pss in maize seeds [11]. In 2023, Scala and colleagues [7] developed and validated a new real-time PCR through an intra-laboratory study. The validation revealed that Scala [7] showed good amplification efficiency and higher analytical sensitivity with respect to Tambong [9] and Pal [10].

The objective of this study was to compare the performance of real-time PCR tests available for the detection of Pss from maize seeds, including an assessment of critical reagents for amplification by Scala [7].

Regarding the PT results, 2 laboratories out of 17 (L2 and L12) achieved an accuracy lower than 100%. L2 uses Pal [10] and AGES [3], which produced false-negative results on samples S2 and S6, both spiked with 10^4^ cfu/mL of Pss. The EPPO PM [3] reported an analytical sensitivity of 95% of agreement at 7 × 10^4^ cfu/mL for these tests. It is hypothesized that the incorrect result is linked to the inability of the AGES [3] to detect Pss in seed samples at 10^4^ cfu/mL of Pss. L12 obtains a false-positive result for sample S3 (10^6^ cfu/mL of Psi) using Tambong [9], Loewe PCR Kit and Pal [10]. The laboratory correctly identifies sample S3 as negative using the Pal [10] method, but in the final interpretation, it does not consider this result. Based on our experience and the results reported in the TPS Pstew-1 report [11], Pal [10] is the only test, among those used by L12, for distinguishing Pss from Psi.

The TPS results prove that Scala [7], validated with two Taq polymerase, is an additional diagnostic test for the specific detection of Pss, suitable for confirmatory and routine analysis of Pss in maize seeds.

In the context of ICL, comparing the results obtained by the OLs within PT and TPS for Pal [10], Tambong [9] and Scala [7] methods, it is possible to argue that Pal [10] and Scala [7] gave the same performance criteria and are equivalent in the Pss diagnosis in maize seeds. Tambong [9] presents a lower diagnostic specificity (83.3%), due to a false positive in the amplification of Psi. For this reason, this method is not advisable for the routine analysis of maize seed samples and should be improved. The participating OLs used different DNA extraction methods, and the results presented in Table 1 indicate that DSE, DSP, and ACC are not affected by the DNA extraction procedure, but rather by the molecular diagnostic test employed for the diagnosis of Pss.

Understanding migration pathways is crucial for monitoring and managing pathogen movement, enabling more proactive disease management and quarantine measures [17]. Genomics data can help identify migration pathways, enabling us to monitor and manage the movement of plant pathogens. This is valuable for plant disease management and quarantine measures [18]. Identifying pathogen origins and transmission pathways during outbreaks, along with understanding how pathogens evolve in response to changing agricultural practices, enhances agricultural productivity [19,20,21].

In this study, we generated high-quality complete genome sequences of Pss strains isolated in Italy from 2015 to 2022 and make them available as a community resource. These resources complement the complete genome of the Pss available in the database. The strains analyzed in this study were isolated in different years and in different locations, but they are very similar in the genome size and in the number of genes identified (Figure 3). The MASH analysis highlighted that all the Pss analyzed in this study are included in the same clade of other Pss collected in Italy and deposited in the database. Among the 53 analyzed genomes, core loci accounted for only 1%, while non-core loci made up 99%. These loci were considered highly stable, which may suggest they evolved as part of the pathogen’s adaptation to a specific niche, such as the maize plant. Agarwal and colleagues [22] report that the number of core genes is about 3500, in line with the results obtained in this work.

## 4. Materials and Methods

### 4.1. Proficiency Test (PT), Test Performance Study (TPS) and ICL (Interlaboratory Comparison)

PT was carried out in Italy in 2022, and 37 sets of samples were prepared: 17 sets were shipped to OLs and 20 sets employed by the CREA-DC organizing laboratory (OrgL) for the verification of homogeneity and stability. To ensure the “blind” execution of the ILC at CREA-DC, the sample panel was prepared by personnel distinct from those conducting the tests. Each set consisted of 10 maize seed extracts, 2 bacterial cell suspensions, 1 positive amplification control representative of Pss CREA-DC 1775 (original name IPV-BO 2766) (PAC1), 2 negative amplification controls with Psi CREA-DC 1923 (original name LMG 2671/NCPPB 1845) (NAC1) and molecular-grade water (NAC2). Details on the sample’s composition are provided in Table 5. The samples consisted of (i) healthy maize seed extracts (*Z. mays*), (ii) healthy maize seed extracts spiked with a known bacterial suspension of Pss CREA-DC 1775, and (iii) a bacterial suspension [10^6^ cfu/mL (colony-forming units/mL)] of Psi or Pan CREA-DC 2059 (original name CFBP 466/NCPPB 441). To artificially spiked samples were added 10^4^ cfu/mL, 10^5^ cfu/mL and 10^6^ cfu/mL Pss bacterial suspensions.

Samples were randomized within each set, and the sets were randomly assigned to each OL. After the randomization process, each sample was labeled with a code consisting of the lab ID and the sample number. One set of samples was shipped to each OL in dry ice. Seventeen OLs, routinely involved in the official Pss analysis, participated in the PT (Table 6), using their internal protocols to perform DNA extraction and amplification. All results are provided qualitatively; therefore, participants were asked to interpret their results as “positive” (pos) or “negative” (neg).

Seventeen OLs that participated in the PT were involved in the TPS, employing the same set of samples (Table 5). For participating in the TPS, the OL participants were also provided with primers and reagents for the execution of the real-time PCR Scala [7], shipped in dry ice. The OLs were divided into two different groups (named Prom and App) according to the different master mixes provided by the organizer for the implementation of the test. Seven OLs (referred to as the App group) performed the test with the 2X Sybr master mix from Applied Biosystems (Thermo Fisher Scientific), and eight OLs (referred to as the Prom group) employed the GoTaq qPCR Master Mix 2x Promega. Scala [7] was performed by the Ols, following the published protocol with a slight modification increasing the amplification cycles from 35 to 40. ICL was conducted using the results provided by the OLs related to the Tambong [9] and Pal [10] real-time PCRs (obtained within the PT), as well as the Scala [7] method, which were performed with two different Taq reagents and obtained within the TPS. All these activities were carried out using the same set of samples.

### 4.2. Plant Material, Bacterial Strains and Sample Preparation

The healthy plant material, *Z. mays* seeds, for sample preparation, originated from commercial batches purchased in Rome, Italy. The *Z. mays* seed extract was prepared as described in EPPO PM [3] and was employed to prepare healthy and spiked samples. The absence of the pathogens Pss and Psi in the maize seed extract was checked by Tambong [9] according to the EPPO PM [3] and Pal [10], modified as reported in the Italian Technical Official Document DTU 27 [23] according to the Valitest project report [11]. The lyophilized bacterial cultures of Pss (strain IPV-BO 2766), Psi (strain CFBP 3614) and Pan (strain CFBP 612/ICMP1850 NCPPB 1846) were revived and cultured in NAG (nutrient agar 0.25% d-glucose). Subsequently, they were grown on King B medium at 27–28 °C for 48 h. Bacterial suspensions were prepared in phosphate buffer (PB 50 mM, pH = 7) for spiking seed samples and in Luria–Bertani medium (LB) (24 h at 28 °C) for bacterial genomic DNA extraction. The bacterial concentrations were spectrophotometrically (DS-11 Fx+, Spectrophotometer-Fluorometer Denovix Inc., Wilmington, DE, USA) measured at OD660 = 0.05, corresponding to approximately 10^8^ cfu/mL. The number of colony-forming units was determined by plating 100 μL of bacterial suspensions on KB medium, incubating them for 48 h at 27–28 °C, and verifying the colony-counting after 2 days.

### 4.3. DNA Extraction

DNA extraction was performed with Gentra Puregene Yeast/Bact. Kit (Qiagen, Venleo, The Netherlands) for bacterial suspension and with the QuickPick™ SML Plant DNA (QRET Technologies Ltd., Turku, Finland) kit, associated with the automated platform KingFisher™ mL Purification System (Thermo Fisher), for healthy and spiked seed extracts.

### 4.4. Homogeneity and Stability 

The samples were tested for their homogeneity and stability according to the EPPO PM [24] before shipment. Ten randomly selected panels of samples were tested for homogeneity, using the DNA extraction protocol described above and Tambong [9] according to the EPPO PM [3]. Short-term stability (7 days) was evaluated using Tambong [9] on three randomly selected panels of samples stored for one week under different temperature conditions (<−15 °C, 2–8 °C, and 25 °C) to simulate sample transportation and storage conditions. After the deadline for result submission (long-term stability, 30 days), three random sample panels stored at −15 °C were analyzed anonymously by Tambong and Pal [9,10]. Each sample was amplified in technical duplicate.

### 4.5. Performance Criteria Evaluation and Outliers

Diagnostic sensitivity (DSE), diagnostic specificity (DSP), and accuracy (ACC) were determined for the PT, TPS and ICL. The percentage of true-negative (TN), false-positive (FP), false-negative (FN) and true-positive (TP) results provided by OLs was calculated. Reproducibility for TPS was obtained according to Langton and colleagues [22], analyzing samples spiked with the pathogen at limit of detection (LOD) (samples S2 and S6).

### 4.6. Genome Analysis

The sequencing and bioinformatic analysis of strains listed in Table 7 were performed by Sequentia Biotech (Barcelona, Spain). In Table 7 are reported the name of the species, the year, and the location of isolation of the samples for which assemblies and annotations were produced in this study. The sequencing was performed by Nanopore (ONT) and Illumina technologies. For the strains CREA-DC 1869 (named Pss_1869), CREA-DC 1870 (named Pss_1870) and CREA-DC 1990 (named Pss_1990), long-read and short-read sequencing data were obtained; for the strains CREA-DC 2123 (named Pss_2123) and CREA-DC 2061 (named Pss_2061), only short-read sequencing was performed. The assemblies CREA-DC 1755 (named Pss_1755) and CREA-DC 1899 (named Pss_1899) were already produced and published by Scala [7]. Illumina reads were trimmed using TRIMMOMATIC (https://github.com/usadellab/Trimmomatic, v0.39, accessed on 12 January 2021) setting minimum quality to 20 and minimum length of the reads to 35 bp. To perform a more detailed pangenome analysis, all the *Pantoea* spp. annotated species present on NCBI (reported in Table 4) were integrated and a mash table (https://github.com/marbl/Mash, v2.3, accessed on 12 February 2021) and a phylogenetic tree (Figure 4) were produced. In order to estimate the genome size of each strain on *P. stewartii*, trimmed reads were used as input for jellyfish (https://github.com/gmarcais/Jellyfish, v2.3.0, accessed on 17 July 2019) to count k-mers, then GenomeScope v2 (http://genomescope.org/genomescope2.0/, accessed on 16 June 2017) was used to graphically represent the genome size estimation.

For the strains Pss_1990, Pss_1870, and Pss_1869, the Bactopia (https://bactopia.github.io/v3.0.0/, v3.0.0, accessed on 19 September 2023) pipeline was utilized to generate genome assemblies. Bactopia is an effective workflow tailored for creating complete prokaryotic genome assemblies. It employs Unicycler (https://github.com/rrwick/Unicycler, v0.5.0, accessed on 21 January 2022) for hybrid assembly and subsequently refines the assembly using short reads with Pilon (https://github.com/broadinstitute/pilon, v1.24, accessed on 11 January 2021). Complete and circularized assemblies were achieved for strains Pss_1990 and Pss_1870. However, for Pss_1869, the assembler could not circularize the genome. Regarding strains Pss_2061 and Pss_2123, a MASH (https://github.com/marbl/Mash, v2.3, accessed on 12 February 2021) distance analysis was carried out using the Illumina reads, comparing them to all other assembled *Pantoea* genomes. This analysis enabled us to pinpoint the closest assembled genome for each isolate. We then utilized these closely related assemblies for reference-guided assembly with SPADES (https://github.com/ablab/spades, v3.15.5, accessed on 17 July 2022). Then, the Redundans (https://github.com/Gabaldonlab/redundans, v2.00, accessed on 27 July 2023) software was applied to close gaps and scaffold the assemblies. In order to evaluate the completeness and the contamination in each sample, we used the software checkM (https://ecogenomics.github.io/CheckM/, v1.2.1, accessed on 20 October 2022). The outputs of this analysis were then used for the genome annotation step.

### 4.7. Genome Annotation, Pangenome and Phylogenomics Analysis

The software Prokka (https://github.com/tseemann/prokka, v1.14.5, accessed on 19 November 2019) is a rapid prokaryotic genome annotation tool and was used to perform the genome annotation of all the genera of *Pantoea* assemblies produced and those downloaded by NCBI (45 assemblies downloaded from NCBI, Table 8). Although the majority of the NCBI genomes were already annotated with PGAP, to perform the pangenome analysis Prokka was used to re-annotate them, producing compatible inputs for the next software packages. The pangenome analysis was performed using PIRATE (https://github.com/SionBayliss/PIRATE, v1.0.5, accessed on 22 July 2022), using as input the annotation files produced for each assembly (the new ones and the NCBI downloaded ones) with several identity thresholds to use for pangenome construction: 50%, 70%, 90%, 95%. Phylogenomics analysis was performed using OrthoFinder (https://github.com/davidemms/OrthoFinder, v2.5.5, accessed on 13 May 2023) using as input files the protein file of each species analyzed (the new assembled Pantoe, those downloaded from NCBI and the output *Pantoea agglomerans*). The species tree was inferred by using the raxml-ng algorithm.

## 5. Conclusions

In this study, we examined two key aspects of Pss knowledge and management. First, through an interlaboratory comparison, we evaluated the performance criteria of various molecular tests for accurately diagnosing the pathogen as well as the reliability of the real-time PCR of Scala [7] when using different reagents. The findings of this study provided valuable insights into molecular diagnostic methods for detecting the pathogen in maize seeds in addition to the available methods [3,4,9,10,11,12,16,25].

The optimization of diagnostic tests by the OLs reduces the risk of false negatives and false positives, thereby preventing the spread of the pathogen through the trade of infected seeds. Specific and timely diagnoses by Ols enable the strengthening of surveillance, reducing the risk of disease spread and enhancing the European and national monitoring systems. This provides important implications for phytosanitary policies and the development of effective prevention and control strategies for Pss in both Europe and Italy.

The data obtained from the ILC can guide import policies, ensuring that seeds from infected areas undergo rigorous diagnostic checks before entering the EU [26,27]. At the European level, phytosanitary authorities coordinate through the network of official laboratories, exchanging information and updates on the most advanced diagnostic methods for more effective management of phytosanitary emergencies and the adoption of common regulations to counter the spread of pathogens.

To further investigate the introduction of this Italian strain and its genomic relationship with strains deposited in the database, we performed whole genome sequencing on newly recovered Pss strains.

Population genetics, which investigates genetic similarities and differences within and between populations, is a powerful tool for uncovering adaptation, speciation, population structure, diversity, and evolution in plant bacterial pathogens [18]. Emergence events can result from various factors, such as adaptation to new hosts or long-distance dissemination of pathogens. Environmental conditions that favour infection and transmission increase the likelihood of epidemic spread, with human activity playing a key role in disease emergence [19]. According to Xu and colleagues [18], genome-wide genetic analysis using next-generation sequencing can explore higher-quality genetic variation compared to traditional multilocus sequencing or a limited number of microsatellites. This approach provides high-resolution insights into the migration and movement patterns of plant pathogen populations.

From a practical perspective, as noted by Estoup and Guillemaud [28], reconstructing invasion routes can help design more effective strategies for preventing invasions. In cases of recurrent introductions, preventive measures may be more cost-effective than eradication or containment efforts [29].

## Figures and Tables

**Table 1:** Value of diagnostic sensitivity (DSE), diagnostic specificity (DSP) and accuracy (ACC) obtained for real-time PCR of Scala [7] with two real-time PCR master mixes (Scala App: 2X Sybr master mix from Applied Biosystems-Thermo Fisher Scientific (Waltham, MA, USA); Scala Prom: GoTaq qPCR Master Mix 2x Promega), for real-time PCR of Pal [10] and Tambong [9], indicated, respectively, as Scala App, Scala Prom, Pal and Tambong. The results for Scala App and Scala Prom were obtained through TPS, while the Pal and Tambong results were derived from PT, both performed on the same set of samples.

Real-Time PCR	DSE%	DSP%	ACC%
Scala App	100	100	100
Scala Prom	100	100	100
Pal	100	100	100
Tambong	100	83.3	100

**Table 2:** FastQC results showing the number of reads per sample before (Raw) and after trimming (Trimmed). Pss = *Pantoea stewartii* subsp. *stewartii*.

Sample	Raw	Trimmed
Pss_1990	4,970,917	4,901,306
Pss_1870	3,853,357	3,812,740
Pss_1869	4,877,111	4,849,311
Pss_2061	5,057,085	5,033,398
Pss_2123	4,793,392	4,764,577

**Table 3:** Quality metrics of the assemblies produced from the *Pantoea stewartii* subsp. *stewartii* strains (Pss).

Sample	Contigs	Dimension bp	Completeness	Contamination
Pss_1990	27	5,442,216	99.34	0.74
Pss_1870	21	5,403,016	99.34	0.74
Pss_1869	58	5,362,941	99.34	0.74
Pss_2061	538	5,171,712	99.34	0.43
Pss_2123	487	5,171,801	99.34	0.25

**Table 4:** Number of genes identified in each strain.

Sample	Genes
Pss_1990	5669
Pss_1870	5607
Pss_1869	5553
Pss_2061	5255
Pss_2123	5295
Pss_1755	5884
Pss_1899	5751

Pss = *Pantoea stewartii* subsp. *stewartii*.

**Table 5:** Sample set prepared for the interlaboratory comparison: 8 samples of maize seed extracts contaminated or not with different concentrations of Pss or Psi or Pan, 2 samples of Pan and Psi strain and 3 controls (PAC1, NAC1 and NAC2). Table indicates sample ID, their composition and related phytosanitary status and the plant matrix used to prepare the samples. Pss = *Pantoea stewartii* subsp. *stewartii* CREA-DC 1775 (original name IPV-BO 2766); Psi = *Pantoea stewartii* subsp. *indologenes* CREA-DC 1923 (original name LMG 2671/NCPPB 1845); Pan = *Pantoea ananatis* CREA-DC 2059 (original name CFBP 466/NCPPB 441), and SE = seed extracts.

Sample ID	Sample Type	Phytosanitary Status	Host
S1	Healthy SE	Negative	Maize seed (*Zea mays*)
S2	SE spiked with 10^4^ cfu/mL of Pss	Positive	Maize seed (*Zea mays*)
S3	Bacterial suspension 10^6^ cfu/mL of Psi	Negative	Bacterial strain
S4	SE	Negative	Maize seed (*Zea mays*)
S5	SE spiked with 10^5^ cfu/mL of Pss	Positive	Maize seed (*Zea mays*)
S6	SE spiked with 10^4^ cfu/mL of Pss	Positive	Maize seed (*Zea mays*)
S7	Bacterial suspension 10^6^ cfu/mL of Pan	Negative	Bacterial strain
S8	SE	Negative	Maize seed (*Zea mays*)
S9	SE spiked with 10^5^ cfu/mL of Pss	Positive	Maize seed (*Zea mays*)
S10	SE spiked with 10^5^ cfu/mL of Pss	Positive	Maize seed (*Zea mays*)
S11	SE	Negative	Maize seed (*Zea mays*)
S12	SE spiked with 10^6^ cfu/mL of Pss	Positive	Maize seed (*Zea mays*)
PAC1	Bacterial DNA (10^6^ cfu/mL of Pss)	Positive	Bacterial strain
NAC1	Bacterial DNA (10^6^ cfu/mL of Psi)	Negative	Bacterial strain
NAC2	Water DEPC (Diethyl pyrocarbonate)	Negative	

**Table 6:** Official laboratories (OLs) participating in the PT and TPS.

Official Laboratories (OLs)
Agenzia Agris, Ussana (SU)
Agenzia Lucana di Sviluppo e di Innovazione in Agricoltura—Centro ricerche metapontum Agrobios ALSIA-CRMA, Metaponto (MT)
Agenzia Settore Agroalimentare delle Marche—SFR Regione Marche, Osimo (AN)
ARSAC, San Marco Argentano (CS)
Centro di Sperimentazione Laimburg (BZ)
CREA-DC Centro difesa e certificazione—Roma
CRSFA, Centro di Ricerca, Sperimentazione e Formazione in Agricoltura “Basile Caramia”, Locorotondo (BA)
DAFNE, Università degli Studi della Tuscia, Dipartimento di Scienze agrarie e forestali, Viterbo (VT)
ERSA, Laboratorio di Fitopatologia e Biotecnologie, Udine (UD)
Fondazione Edmund Mach, San Michele all’Adige (TN)
Laboratorio Fitopatologico Regione Campania, Napoli (NA)
Laboratorio Fitopatologico Regione Emilia-Romagna, Bologna (BO)
Laboratorio fitosanitario e settore fitosanitario e servizi tecnico-scientifici, Direzione Agricoltura e cibo, Regione Piemonte, Torino (TO)
Laboratorio SFR Regione Lombardia, Vertemate con Minoprio (MI)
Laboratorio Fitopatologico Regione Liguria, Genova (GE)
Regione Toscana SFR e di vigilanza del controllo agroforestale—Laboratorio Fitopatologico Regionale, Pistoia (PT)
Regione Veneto Unità organizzativa fitosanitario, Buttapietra (VR)

**Table 7:** Species name, year and location of isolation and sample name. Assembly reports the reference of the assembly.

Species Name	Year	Location	Sample	Assembly
*Pantoea stewartii* subsp. *stewartii*	2015	Italy, Emilia Romagna	Pss_1869	This study
*Pantoea stewartii* subsp. *stewartii*	2015	Italy, Emilia Romagna	Pss_1870	This study
*Pantoea stewartii* subsp. *stewartii*	2018	Italy, Emilia Romagna	Pss_1990	This study
*Pantoea stewartii* subsp. *stewartii*	2021	Italy, Emilia Romagna	Pss_2061	This study
*Pantoea stewartii* subsp. *stewartii*	2022	Italy, Emilia Romagna	Pss_2123	This study

**Table 8:** Metadata related to the *Pantoea stewartii* assemblies used for the MASH, phylogenesis and pangenome analysis. Columns report the accession code, the accession name, the organism, the strain and the date of submission.

Code	Assembly Name	Organisms	Strain	Date of Submission
GCF_011044475.1	ASM1104447v1	*Pantoea stewartii*	ZJ-FGZX1	1 March 2020
GCF_025765915.1	ASM2576591v1	*Pantoea stewartii*	HR3-48	20 October 2022
GCF_002082215.1	ASM208221v1	*Pantoea stewartii* subsp. *stewartii*	DC283	10 April 2017
GCF_000757405.2	LMG2632assem4	*Pantoea stewartii* subsp. *indologenes*	LMG 2632	23 September 2014
GCF_008801695.1	ASM880169v1	*Pantoea stewartii* subsp. *stewartii*	CCUG 26359	1 October 2019
GCF_029433915.1	ASM2943391v1	*Pantoea stewartii*	ICMP 10132	27 March 2023
GCF_001310285.1	Pantoea_stewartii_A206_1.0	*Pantoea stewartii*	A206	13 October 2015
GCF_013277595.1	ASM1327759v1	*Pantoea stewartii*	626	4 June 2020
GCF_025599245.1	ASM2559924v1	*Pantoea stewartii*	ST25	5 October 2022
GCF_014218605.1	ASM1421860v1	*Pantoea stewartii*	NRRL B-133	17 August 2020
GCF_000803205.1	ASM80320v1	*Pantoea stewartii*	M073a	17 December 2014
GCF_029991035.1	ASM2999103v1	*Pantoea stewartii*	C10109_Jinnung	12 May 2023
GCF_001310295.1	Pantoea_stewartii_S301_1.0	*Pantoea stewartii*	S301	13 October 2015
GCF_001476375.1	ASM147637v1	*Pantoea stewartii*	RSA36	22 December 2015
GCF_001476355.1	ASM147635v1	*Pantoea stewartii*	NS381	22 December 2015
GCF_000786255.1	ASM78625v1	*Pantoea stewartii*	M009	23 November 2014
GCF_001476795.1	ASM147679v1	*Pantoea stewartii*	RSA30	22 December 2015
GCF_001477215.1	ASM147721v1	*Pantoea stewartii*	RSA13	22 December 2015
GCF_000248395.1	ASM24839v2	*Pantoea stewartii* subsp. *stewartii*	DC283	22 February 2012
GCF_030370575.1	ASM3037057v1	*Pantoea stewartii* subsp. *indologenes*	LMG 2671	28 June 2023
GCF_017052135.1	ASM1705213v1	*Pantoea stewartii* subsp. *indologenes*	PNA 14-12	27 February 2021
GCF_017052195.1	ASM1705219v1	*Pantoea stewartii* subsp. *indologenes*	PNA 14-11	27 February 2021
GCF_017051895.1	ASM1705189v1	*Pantoea stewartii* subsp. *indologenes*	NCPPB 2275	27 February 2021
GCF_017051975.1	ASM1705197v1	*Pantoea stewartii* subsp. *indologenes*	PANS 07-10	27 February 2021
GCF_017052015.1	ASM1705201v1	Pantoea stewartii subsp. *indologenes*	PANS 07-12	27 February 2021
GCF_017052375.1	ASM1705237v1	*Pantoea stewartii* subsp. *indologenes*	PNA 14-9	27 February 2021
GCF_017052175.1	ASM1705217v1	*Pantoea stewartii* subsp. *indologenes*	PNA 15-2	27 February 2021
GCF_017051945.1	ASM1705194v1	*Pantoea stewartii* subsp. *indologenes*	PANS 99-15	27 February 2021
GCF_017051805.1	ASM1705180v1	*Pantoea stewartii* subsp. *indologenes*	NCPPB 2281	27 February 2021
GCF_017051935.1	ASM1705193v1	*Pantoea stewartii* subsp. *indologenes*	PANS 07-14	27 February 2021
GCF_017052095.1	ASM1705209v1	*Pantoea stewartii* subsp. *indologenes*	PANS 07-4	27 February 2021
GCF_017052115.1	ASM1705211v1	*Pantoea stewartii* subsp. *indologenes*	PANS 07-6	27 February 2021
GCF_017051815.1	ASM1705181v1	*Pantoea stewartii* subsp. *indologenes*	NCPPB 2282	27 February 2021
GCF_017051845.1	ASM1705184v1	*Pantoea stewartii* subsp. *indologenes*	NCPPB 1562	27 February 2021
GCF_030144305.1	ASM3014430v1	*Pantoea stewartii* subsp. *indologenes*	SCHI0154.S.1	31 May 2023
GCF_017051875.1	ASM1705187v1	*Pantoea stewartii* subsp. *indologenes*	NCPPB 1877	27 February 2021
GCF_030370585.1	ASM3037058v1	*Pantoea stewartii* subsp. *stewartii*	IPV-BO 2766	28 June 2023
GCF_030370545.1	ASM3037054v1	*Pantoea stewartii* subsp. *stewartii*	49474.1 /18	28 June 2023
GCF_010273335.1	ASM1027333v1	*Pantoea stewartii* subsp. *stewartii*	MS1	6 February 2020
GCF_030064655.1	ASM3006465v1	*Pantoea stewartii*		23 May 2023
GCA_030336395.1	ASM3033639v1	*Pantoea stewartii* subsp. *indologenes*	LMG 2630	22 June 2023
GCA_030336485.1	ASM3033648v1	*Pantoea stewartii* subsp. *stewartii*	34258.2/15	22 June 2023
GCA_030370555.1	ASM3037055v1	*Pantoea stewartii* subsp. *stewartii*	49472.2 /18	28 June 2023
GCA_030336515.1	ASM3033651v1	*Pantoea stewartii* subsp. *stewartii*	34596.1/15	22 June 2023
GCA_030370565.1	ASM3037056v1	*Pantoea stewartii* subsp. *stewartii*	CREA_DC_1788	28 June 2023

## Data Availability

SRA https://www.ncbi.nlm.nih.gov/sra/PRJNA1258867, accessed on 5 May 2025; Mendeley https://data.mendeley.com/datasets/2xg852nsh8/1, accessed on 5 May 2025.

## Supplementary Materials

The following supporting information can be downloaded at: https://www.mdpi.com/article/10.3390/plants14101470/s1, Table S1 Results obtained from the evaluation of the homogeneity of the samples prepared for the PT an TPS. Ten panels were tested for each sample in technical duplicate using real-time PCR (Tambong et al., 2008) [9]. The phytosanitary status of each sample, the maximum and minimum Cq values obtained for each sample, the mean Cq values, and the respective coefficient of variation values are reported. Legend: Cq = cycle threshold, min = minimum Cq value, max = maximum Cq value, CV = coefficient of variation, NA = not amplified, ES = seed extract. Pss = *Pantoea stewartii* subsp. *stewartii*; Psi = *Pantoea stewartii* subsp. *indologenes*; Pan = *Pantoea ananatis*. Table S2 Results obtained for the evaluation of the stability (mid-term and long-term stability) of the samples prepared for the PT and TPS. The mid-term is evaluated at 7 days at different temperature. The long-term stability is at 30 days at T-15 °C. Three panels were tested for each sample in technical duplicate using real-time PCR (Tambong et al., 2008) [9]. The phytosanitary status of each sample, the maximum and minimum Cq values obtained for each sample, the mean Cq values, and the respective coefficient of variation values are reported. Legend: Cq = cycle threshold, min = minimum Cq value, max = maximum Cq value, CV = Coefficient of Variation, NA = not amplified, ES = seed extract. Pss = *Pantoea stewartii* subsp. stewartii; Psi = *Pantoea stewartii* subsp. indologenes; Pan = *Pantoea ananatis*. Table S3 Results obtained from the evaluation of the stability (long-term stability) of the samples prepared for the PT and TPS. The long-term stability is at 30 days at T-15 °C. Two panels were tested for each sample in technical duplicate using real-time PCR (Pal et al., 2019) [10]. The phytosanitary status of each sample, the maximum and minimum Cq values obtained for each sample, the mean Cq values, and the respective coefficient of variation values are reported. Legend: Cq = cycle threshold, min = minimum Cq value, max = maximum Cq value, CV = Coefficient of Variation, NA = not amplified, SE = seed extract. Pss = Pantoea stewartii subsp. stewartii; Psi = Pantoea stewartii subsp. indologenes; Pan = Pantoea ananatis. Table S4 Qualitative results obtained from the PT participants. The column “Lab ID” shows the number associated with each laboratory participating in the PT. The PT samples are listed as S (sample) and a number from 1 to 12. For each sample, the positivity or negativity status is indicated. The table reports the expected PAC results for Pantoea stewartii subsp. stewartii (PAC Pss) and Pantoea stewartii subsp. indologenes (PAC Pind), as well as the compliant NAC for each participant. For each participant, the percentage values of true positives and true negatives are reported, along with the calculated accuracy percentage, as outlined in PM 7/122 (2) [24]. Legend: pos = positive, neg = negative, ACC = accuracy.

## Abbreviations

Accuracy (ACC); Diagnostic sensitivity (DSE); Diagnostic specificity (DSP); European Plant Protection Organization (EPPO); Interlaboratory comparisons (ILC); Official laboratory (OL); *Pantoea ananatis* (Pan); *Pantoea indologenes* (Psi); *Pantoea stewartii* subsp. *stewartii* (Pss); Proficiency Test (PT); Seed extracts (SE); Test Performance Study (TPS).
