# Supplementary material for: Pantoea stewartii subsp. stewartii an Inter-Laboratory Comparative Study of Molecular Tests and Comparative Genome Analysis of Italian Strains
Source: Plants (Basel). 2025 May 14;14(10):1470. doi: 10.3390/plants14101470 (PMC12114735; doi:10.3390/plants14101470)
Supplement: Supplementary file 1 [file plants-14-01470-s001.zip › plants-3589884-supplementary.pdf]

Supplementary Materials

Table S1 Results obtained from the evaluation of the homogeneity of the samples prepared for the PT an TPS. Ten panels were tested for each sample in technical duplicate using real-time PCR (Tambong et al., 2008)[9]. The phytosanitary status of each sample, the maximum and minimum Cq values obtained for each sample, the mean Cq values, and the respective coefficient of variation values are reported. Legend: Cq = cycle threshold, min = minimum Cq value, max = maximum Cq value, CV = coefficient of variation, NA = not amplified, ES = seed extract. Pss = *Pantoea stewartii* subsp. *stewartii;* Psi = *Pantoea stewartii* subsp. *indologenes;* Pan = *Pantoea ananatis*

Sample ID	Sample types	Sanitary Status	Real-time PCR Tambong et al., (2008)[9]	
			min-max (Cq)	Average (Cq)±CV
S1-S4-S8-S11	Healthy SE	Neg	NA	
S2-S6	SE + 10^4 cfu/ml Pss	Pos	29,55 - 30,57	30,16 ± 0,009
S5- S9- S10	SE +10^5 cfu/ml Pss	Pos	27,30 - 28,99	28,20 ± 0,015
S12	ES+10^6 Pss	Pos	20,59 - 25,35	24,38 ± 0.035
S7	Pan 10^6 cfu/ml	Neg	NA	
S3	Psi 10^6 cfu/ml	Neg	23,19 - 24,37	23,67 ± 0.010
PAC	Pss 10^6 cfu/ml	Pos	28.60-29.36	29,14 ± 0.005
PAC	Psi 10^6 cfu/ml	Pos	27.15-27.46	27.30± 0.003
NAC	acqua DEPC	Neg	NA	

Table S2 Results obtained for the evaluation of the stability (mid-term and long-term stability) of the samples prepared for the PT and TPS .The mid-term is evaluated at 7 days at different temperature. The long-term stability is at 30 days at T-15°C. Three panels were tested for each sample in technical duplicate using real-time PCR (Tambong et al., 2008)[9]. The phytosanitary status of each sample, the maximum and minimum Cq values obtained for each sample, the mean Cq values, and the respective coefficient of variation values are reported. Legend: Cq = cycle threshold, min = minimum Cq value, max = maximum Cq value, CV = Coefficient of Variation, NA = not amplified, ES = seed extract. Pss = *Pantoea stewartii* subsp. stewartii; Psi = *Pantoea stewartii* subsp. indologenes; Pan = *Pantoea ananatis*

Sample ID	Sample types	Sanitary Status	Real-time Tambong et al., (2008)[9]							
			7 days T-15°C		7 days T 4°C		7 days T 25°C		30 days T-15°C	
			min-max (Cq)	average (Cq)±CV	min-max (Cq)	average (Cq)±CV	min-max (Cq)	average (Cq)±CV	min-max (Cq)	average (Cq)±CV
S1-S4-S8-S11	Healthy SE	Neg	NA		NA		NA		NA	
S2-S6	SE + 10^4 cfu/ml Pss	Pos	30,38-31,35	30,92±0.01	31,50-34,57	32,79±0.03	26,96-30,59	28,79±0.05	31,28-33,08	32,64±0.02
S5- S9-S10	SE +10^5 cfu/ml Pss	Pos	28.60-29.80	29.30±0.02	29,36-31,44	30,23±0.03	22,87-26,87	25,00±0.06	30.19-30.46	31.21±0.07
S12	SE +10^6 Pss	Pos	25.16-25.63	25.46±0.01	25.81-26.98	26,14±0.01	28,23-32,20	29,95±0.05	26.63-27.20	26.88±0.01
S7	Pan 10^6 cfu/ml	Neg	NA		NA		NA	NA	NA	NA
S3	Psi 10^6 cfu/ml	Neg	22.92-23.59	23.14±0.01	23,80-24.49	24,25±0.01	23.58-24.87	24,24±0.02	24.99-24.45	24.23±0.01
PAC	ES+10^6 Pss	Pos	27.50-28.19	27.77±0.01	27.18-27.60	27,39±0.01	25.90-27.53	26,83±0.02	27.63-28.02	27.81±0.01
PAC	Psi 10^6 cfu/ml	Neg	25.79-26.24	26.07±0.00	25.46-25.84	25.67±0.00	24.27-24.81	24.49±0.01	25.73-26.16	25.93±0.01
NAC	H_20 DEPC	Neg	NA		NA		NA		NA	

Table S3 Results obtained from the evaluation of the stability (long-term stability) of the samples prepared for the PT and TPS. The long-term stability is at 30 days at T-15°C. Two panels were tested for each sample in technical duplicate using real-time PCR (Pal et al., 2019)[10]. The phytosanitary status of each sample, the maximum and minimum Cq values obtained for each sample, the mean Cq values, and the respective coefficient of variation values are reported. Legend: Cq = cycle threshold, min = minimum Cq value, max = maximum Cq value, CV = Coefficient of Variation, NA = not amplified, SE = seed extract. Pss = Pantoea stewartii subsp. stewartii; Psi = Pantoea stewartii subsp. indologenes; Pan = Pantoea ananatis

ID sample	Host plant	Status	Real-time Pal et al. (2019)[10]	
			30 days T-15°C	
			min-max (Cq)	average (Cq)±CV
**S1-S4-S8-S11**	SE	Neg	NA	
**S2-S6**	SE + 10^4 cfu/ml Pss	Pos	36,09-37,82	36,96±0.02
**S5-S9-S10**	SE +10^5 cfu/ml Pss	Pos	35.18-37.21	35.83±0.02
**S12**	SE+10^6 Pss	Pos	31.50-32.21	31.86±0.02
**S7**	Pan 10^6 cfu/ml	Neg	NA	NA
**S3**	Psi 10^6 cfu/ml	Neg	NA	NA
**PAC**	SE+10^6 Pss	Pos	31.99-32.86	32.43±0.02
**PAC**	Psi 10^6 cfu/ml	Neg	NA	NA
**NAC**	H_20 DEPC	Neg	NA	

Table S4 Qualitative results obtained from the PT participants. The column "Lab ID" shows the number associated with each laboratory participating in the PT. The PT samples are listed as S (sample) and a number from 1 to 12. For each sample, the positivity or negativity status is indicated. The table reports the expected PAC results for Pantoea stewartii subsp. stewartii (PAC Pss) and Pantoea stewartii subsp. indologenes (PAC Pind), as well as the compliant NAC for each participant. For each participant, the percentage values of true positives and true negatives are reported, along with the calculated accuracy percentage, as outlined in PM 7/122 (2)[26]. Legend: pos = positive, neg = negative, ACC = accuracy.

LAB ID	S1	S2	S3	S4	S5	S6	S7	S8	S9	S10	S11	S12	PAC Pss	PAC Pind	NAC	% True Pos	% True Neg	% ACC
	**Status samples**																	
	**neg**	**pos**	**neg**	**neg**	**pos**	**pos**	**neg**	**neg**	**pos**	**pos**	**neg**	**pos**						
1	neg	pos	neg	neg	pos	pos	neg	neg	pos	pos	neg	pos	pos	neg	neg	100	100	100
2	neg	neg	neg	neg	pos	neg	neg	neg	pos	pos	neg	pos	pos	neg	neg	66,7	100	83,3
4	neg	pos	neg	neg	pos	pos	neg	neg	pos	pos	neg	pos	pos	neg	neg	100	100	100
5	neg	pos	neg	neg	pos	pos	neg	neg	pos	pos	neg	pos	pos	neg	neg	100	100	100
6	neg	pos	neg	neg	pos	pos	neg	neg	pos	pos	neg	pos	pos	neg	neg	100	100	100
7	neg	pos	neg	neg	pos	pos	neg	neg	pos	pos	neg	pos	pos	neg	neg	100	100	100
8	neg	pos	neg	neg	pos	pos	neg	neg	pos	pos	neg	pos	pos	neg	neg	100	100	100
9	neg	pos	neg	neg	pos	pos	neg	neg	pos	pos	neg	pos	pos	neg	neg	100	100	100
10	neg	pos	neg	neg	pos	pos	neg	neg	pos	pos	neg	pos	pos	neg	neg	100	100	100
11	neg	pos	neg	neg	pos	pos	neg	neg	pos	pos	neg	pos	pos	neg	neg	100	100	100
12	neg	pos	pos	neg	pos	pos	neg	neg	pos	pos	neg	pos	pos	neg	neg	100	83,3	91,7
13	neg	pos	neg	neg	pos	pos	neg	neg	pos	pos	neg	pos	pos	neg	neg	100	100	100
14	neg	pos	neg	neg	pos	pos	neg	neg	pos	pos	neg	pos	pos	neg	neg	100	100	100
15	neg	pos	neg	neg	pos	pos	neg	neg	pos	pos	neg	pos	pos	neg	neg	100	100	100
16	neg	pos	neg	neg	pos	pos	neg	neg	pos	pos	neg	pos	pos	neg	neg	100	100	100
18	neg	pos	neg	neg	pos	pos	neg	neg	pos	pos	neg	pos	pos	neg	neg	100	100	100
